# Optimizing PCR amplification of GC-rich nicotinic acetylcholine receptor subunits from invertebrates

**DOI:** 10.1016/j.bbrep.2025.102052

**Published:** 2025-05-15

**Authors:** Muhammad Tanveer Khan, Coralie Belgrano, Lucien Rufener, Tor Einar Horsberg, Marit Jorgensen Bakke

**Affiliations:** a Norwegian University of Life Sciences, Faculty of Veterinary Medicine, Aas, Norway; b Invenesis Sarl, Rue de Neuchâtel 15a, St-Blaise, Switzerland

**Keywords:** Nicotinic acetylcholine receptor, GC-rich gene, Betaine, DMSO, DNA polymerases

## Abstract

Polymerase chain reaction (PCR) is a widely used molecular biology technique for amplifying specific DNA sequences. However, amplifying templates with a high GC content (>60 %) poses challenges owing to strong hydrogen bonds and secondary structure formation, hindering DNA polymerase activity and primer annealing. Given these challenges, our study focuses on refining PCR protocols for the nicotinic acetylcholine receptor subunits, pivotal for understanding signal transduction in various organisms and potential important drug targets. We optimized the PCR protocol to efficiently amplify the beta1 and alpha1 subunits of the nicotinic acetylcholine receptor from *Ixodes ricinus* (*Ir-nAChRb1*) and *Apis mellifera* (*Ame-nAChRa1*). *Ir-nAChRb1* and *Ame-nAChRa1* have open reading frames of 1743 and 1884 bp, respectively, with overall GC contents of 65 % and 58 %. Various DNA polymerases and organic additives have been evaluated at different annealing temperatures. The tailored protocol incorporated organic additives, such as DMSO and betaine, increased enzyme concentration, and adjusted annealing temperatures. This study demonstrates the importance of a multipronged approach involving various organic molecules, DNA polymerases, PCR conditions, and primer adjustments to overcome the challenges of amplifying GC-rich sequences.

## Highlights

• PCR amplification of genes with a high GC content (>60 %) is challenging.
• The amplification of two nAChR subunits from invertebrates presents a challenge.
• Organic additives, primer lengths, and polymerases help amplify GC-rich targets.
• A study to maximize the GC-rich target amplification.

## 1 Introduction

Polymerase chain reaction (PCR), developed by Kary Mullis in 1983, has become an indispensable technique in molecular biology to produce multiple copies of specific deoxyribonucleic acid (DNA) *in vitro*. A widely used technique in genome sequencing, gene expression analysis, molecular genetics, and the diagnosis of infectious diseases, PCR can be challenging to amplify templates in which there is a long, short tandem region and a high GC/AT content [[1], [2], [3], [4]]. Sequences with a high GC content (>60 %) pose difficulties owing to their higher melting temperatures under standard PCR conditions. Strong hydrogen bonds between guanine and cytosine bases, along with the formation of secondary structures such as hairpins, knots, and tetraplexes, hinder DNA polymerase activity or primer annealing, resulting in PCR failure or truncated PCR products [5]. Additionally, primers containing a high GC content may produce misprimed products during PCR [6].

To overcome these challenges, various organic molecules are routinely integrated into the amplification process. GC-rich genes exhibit enhanced amplification when compounds such as dimethyl sulfoxide (DMSO), betaine, formamide, and 7-deaza-dGTP are added to the PCR mixture [2,[7], [8], [9], [10], [11], [12], [13], [14], [15]]. The amplification efficiency can be further improved by combining two or three additives [9,10]. These additives aid in resolving complex secondary structures in GC-rich regions, thereby reducing primer or template melting temperatures [10,16]. Additionally, sodium hydroxide (NaOH) treatment effectively denatured highly GC-rich templates, significantly enhancing PCR amplification [17]. Besides additives, utilizing different commercially available DNA polymerases, primer modifications, hot-start PCR, touchdown, and slowdown PCR strategies can also improve amplification efficiency [2,6,18,19]. However, a single approach may not provide universal GC-rich region amplification, necessitating a multipronged approach involving multiple organic molecules, DNA polymerases, PCR conditions, and primer adjustment.

Nicotinic acetylcholine receptors (nAChRs) are ligand-gated ion channels in the cys-loop family that are crucial for chemoelectrical signal transduction and modulation in the nervous system and neuromuscular junctions [20,21]. The nAChRs are pentameric transmembrane proteins, expressed in both vertebrates and invertebrates. The five subunits forming the complex around a central pore can be either the same subunit, resulting in a homo-pentameric receptor, or a combination of different subunits, resulting in a hetero-pentameric receptor. Different combinations of different subunits result in a broad variety of nAChR subtypes [[22], [23], [24]] Recently, we amplified and cloned nAChR subunits from the European honeybee (*Apis mellifera*) and the castor bean tick (*Ixodes ricinus*). Two subunits exhibited regions of high GC content, rendering the PCR unsuccessful with standard procedures. This was the case for the alpha1 subunit from *Apis mellifera* (*Ame-nAChRa1*) and the beta1 subunit from *Ixodes ricinus* (*Ir-nAChRb1*).

This study aimed to optimize the PCR protocol to effectively amplify these GC-rich sequences by combining various organic molecules, DNA polymerases, PCR conditions, and carefully selecting primer adjustments.

## 2 Materials and methods

### 2.1 Biological material and RNA extraction

Adult female ticks (*I. ricinus*) were purchased from IS Insect Services (Germany). Upon receipt, tick samples were placed on dry ice for rapid freezing and stored at −80 °C until further processing. RNA was extracted from adult ticks using a RNeasy Micro Kit (Qiagen, Germany) and phenol-chloroform. Ticks were homogenized in two steps. Female ticks (n = 4) were initially placed in a 1.5 ml Eppendorf tube containing 1 ml TRIzol reagent (Sigma-Aldrich), a monophasic solution of phenol and guanidine isothiocyanate for the isolation of high-quality RNA, and the ticks were crushed manually with a pestle. To prevent overheating, the samples were placed intermittently on dry ice during homogenization for five to 10 min. The manually homogenized samples were transferred to 2 ml tubes with a 5 mm stainless steel bead undergoing automated homogenization two to three times at 5000–6000 revolutions per minute (rpm) for 30 s (Precellys, Bertin Technologies). After the completion of the homogenization step, 0.2 ml of chloroform (Sigma-Aldrich), which promotes phase separation for high-quality RNA, was added to the homogenized samples and incubated for 3 min. Samples were centrifuged for 15 min at 12000×*g* at 4 °C for phase separation. The aqueous phase was removed from the centrifuged samples, and RNA was extracted using the RNeasy Micro Kit (Qiagen, Germany) following the manufacturer's instructions. The concentration and quality of the eluted RNA were assessed using spectrophotometry (Epoch, BioTek) and 1 % RNA agarose gel electrophoresis in 1X TBE (Tris-borate-EDTA) buffer.

Living adult bees were obtained from beekeepers and shock-frozen in liquid nitrogen. For RNA extraction, individual bees were homogenized in TRIzol reagent using a Tissue Ruptor (Invitrogen) and processed according to the manufacturer's instructions. To remove DNA contamination, the RNA samples were treated using a TURBO DNA-free kit (Ambion). Total RNA was extracted from individual bee heads using a RNeasy Mini Kit (Qiagen, Germany). The concentration and quality of the eluted RNA were assessed using NanoDrop (Thermo Scientific) and 1 % RNA agarose gel electrophoresis using 1X TBE running buffer.

### 2.2 Complementary DNA (cDNA) synthesis

For ticks, the AffinityScript qPCR cDNA Synthesis Kit (Agilent, USA) was used to reverse-transcribe 1 μg of RNA using OligodT and Random hexamer primers. The resulting cDNA was diluted (1:2) in nuclease-free water and stored at −20 °C until further use. Betaine (1 M) and DMSO (5 %), individually or in combination, were incorporated during cDNA synthesis as needed.

For bees, a total of 1 μg total RNA (DNase-treated) was reverse-transcribed to cDNA using a (dT)30 primer and SuperScript III Reverse Transcriptase (Invitrogen, Carlsbad, CA) for 1 h at 55 °C. The reaction was conducted in a total volume of 20 μl. The resulting cDNA was stored at −20 °C until further use.

### 2.3 Primers for PCR amplification

Full-length mRNA sequences of *Ir-nAChRb1* and *Ame-nAChRa1* were retrieved from the National Center for Biotechnology Information (NCBI) (accession numbers MZ027281.1 and XM_026442626.1, respectively. Primers used for PCR and sequencing were designed using Primer-BLAST [25] for *Ir-nAChRb1* and Primer3 software (Primer3 Input) for *Ame-nAChRa1* (Table 1). The annealing temperatures for the PCR reactions were determined based on the manufacturer's recommendations or calculated using *Tm* calculators (www.thermofisher.com/tmcalculator).

**Table 1:** Primers used for PCR reactions.

Name	Sequence (5'-3')	Gene
NheI_Ame-AChRa1F1	GGCGGCTAGCGTCTAGGTTGGGCGGATTG	*Ame-nAChRa1*
XhoI_Ame-AChRa1R1	GGCGCTCGAGGGATCGTCGACTAGTCCTCCT	
NheI_Ame-AChRa1F2	GGCGGCTAGCACGATGCTTAGGGATGCTTG	
Ame-nAChR-a1_F5	ATGGCGACGGCCATTTCC	
Ame-nAChR-a1_R5	CGGCAGTGGGAGCGAGGGTA	
ClaI_Ir_nAChRb1-F	ATTCATCGATACCATGGGCGCAGCAGCGGC	*Ir-nAChRb1*
AfiII_Ir_nAChRb1-R	ATGGCTTAAGTTCACGTGGGCTTGCCGCGGT	
Ir-nAChRb1-F1	GAGGAGCAGAGCAGCGAG	
Ir-nAChRb1-R1	GATGCTTGTGGGTTACTCGCT	
Ir-nAChRb1-DF1	ACCTGCTGTTCACTTTCCTC	
Ir-nAChRb1-DR1	ATCACCATGGCCACGTACTT	

### 2.4 Polymerases and PCR conditions

SuperScript IV One-Step RT-PCR System was purchased from Invitrogen (12594025) and includes SuperScript IV Reverse Transcriptase (RT) along with high-fidelity Platinum SuperFi DNA Polymerase, facilitating cDNA synthesis and the efficient amplification of low-abundant and longer genes (13.8 Kb) directly from RNA in a single reaction. Phusion™ High–Fidelity (F530S) and Platinum™ SuperFi™ (12351010) were purchased from Invitrogen. These DNA polymerases have proofreading activity and are accompanied by GC enhancers and buffers, designed to amplify GC-rich targets with low error rates. PCR reactions were performed in a 25-μl volume following the supplier's instructions. The enzyme concentration was increased to 0.03 U/μl when the enhancers DMSO and betaine were added. A 2-step PCR protocol was used when the annealing step required 72 °C, and the annealing and extension steps were combined. Other DNA polymerases were also tested, based on their easy availability and different properties. Dream Taq DNA polymerase (EP0711) and Hot-start Taq-based polymerases such as AccuPrime™ Taq DNA Polymerase (12339016) were purchased from Thermo Scientific and Invitrogen, respectively, and the manufacturer's guidelines were followed for the PCR reactions. The details of the thermocycler conditions are listed in Table 2.

**Table 2:** Thermocycler conditions during PCR.

Steps	SuperScript		Phusion/Platinum		AccuPrime		Dream Taq		
	Temp	Time	Temp	Time	Temp	Time	Temp	Time	
Reverse transcription	50-60	10 min	–	–	–	–	–	–	
Initial denaturation	98	3 min	98	3 min	94	2 min	95	3 min	
Denaturation	98	10 Sec	98	30 s	94	30 s	95	30 s	35 Cycles
Annealing	55-70	10 Sec	57-67.5	10 s	55-60	20 s	55-57	30 s	
Extension	72	1 min	72	1 min	68	2 min	72	1 min	
Final Extension	72	5 min	72	5 min	68	5 min	72	10 min	
Holding	4	∞	4	∞	4	∞	4	∞	

Temperature is shown in °C. SuperScript, SuperScript IV One-Step RT-PCR; Phusion, Phusion™ High–Fidelity; Platinum, Platinum™ SuperFi™; AccuPrime, AccuPrime™ Taq DNA Polymerase; DreamTaq, Dream Taq DNA polymerase.

The concentrations of DMSO and betaine used in either cDNA or PCR reactions are presented in detail in the results section. All PCR products were subjected to 1 % agarose gel electrophoresis in the presence of 1X Tris-acetate-EDTA (TAE) buffer. PCR products with the expected amplicon lengths were gel-cut and purified using a MinElute PCR Purification Kit (Qiagen, Germany). Purified PCR products were sequenced using LightRun (Eurofins Genomics, Germany).

### 2.5 Cloning of *Ir-nAChRb1* and *Ame-nAChRb1*

The full-length open reading frame (ORF) of *Ir-nAChRb1* was cloned into a pT7TS-rich transcription vector, *which introduced X. laevis β-globin untranslated cDNA to the 5′ and 3′ ends of the gene* using a restriction enzyme-based approach. For restriction enzyme-based cloning, primers were designed using Primer-BLAST by introducing *ClaI* and *AflII* restriction enzyme sites in the forward and reverse primers, respectively. The insert and vector were double-digested, followed by ligation (at a ratio of 2:1) using T4 DNA Ligase (NEB: M0202S). The ligated mixture was transformed into MAX Efficiency™ DH5α Competent Cells (Invitrogen, USA) following the manufacturer's protocol. The plasmids were purified using a ZymoPURE Plasmid Miniprep Kit (Zymo) and sequenced using LightRun (Eurofins Genomics, Germany).

*For Ame-nAChR-a1, the PCR products were digested with NheI and XhoI restriction enzymes. They were then analyzed on 1 % agarose gels, excised, gel-purified (QIAquick Gel Extraction Kit; Qiagen*, Germany*), and cloned into the pT7TS-rich transcription vector using T4 DNA ligase (Thermo Scientific). Plasmid DNA was purified using the Plasmid Plus Midi Kit (Qiagen, Germany), and at least three clones of each construct were sequenced using pT7TS-specific forward and reverse primers at Microsynth (*Microsynth AG - Microsynth - CH*).*

### 2.6 Bioinformatics

Sequence assembly was performed using Benchling (https://benchling.com) or *Geneious v5.6.7 (*https://www.geneious.com*),* and alignment analysis was conducted using BioEdit [26]. GC content in a DNA sequence was calculated using Webgenetics (Webgenetics - online bioinformatics tools for sequence analysis). For nucleotide sequences, a nucleotide blast was made online (NCBI) against the nucleotide collection (NT*).*

## 3 Results and discussion

**Fig. 1:**
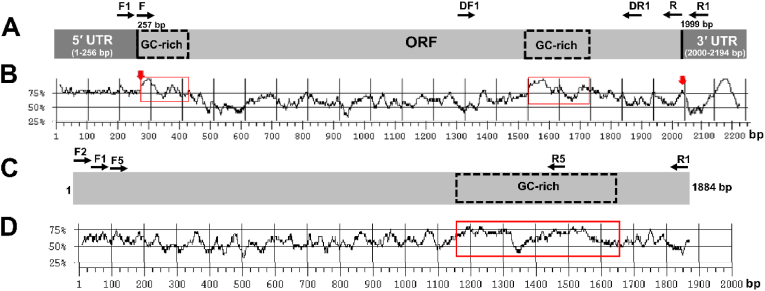
The GC contents of the two nAChR subunit genes are presented. An overview of the open reading frame (ORF) of *Ir-nAChRb1* and the coding sequence of *Ame-nAChRa1* is outlined in panels A and C, respectively. The 5' and 3' UTRs are represented by dark grey boxes, and the ORFs are highlighted in light grey. GC content greater than 80 % is indicated by dotted boxes. The black arrows indicate the positions of the primers used to amplify the full ORF or a short segment of the ORF. The distribution of GC content along the mRNA sequences is indicated for *Ir-nAChRb1* and *Ame-nAChRa1* in panels B and D, respectively. Start and stop codons are indicated with red arrows and GC content above 80 % is highlighted with red boxes.

Transcripts with a GC content exceeding 65 % challenge standard PCR amplification owing to the formation of secondary structures that impede template denaturation and primer annealing. This was confirmed by our attempts to amplify the complete ORFs of *Ir-nAChRb1* and *Ame-nAChR-a1* using a single PCR reaction for cloning into a pT7TS-rich transcription vector. Full-length *Ir-nAChRb1* spans 2194 nucleotides, comprising 1743 bp of ORF, 256-bp 5'-untranslated region (UTR), and a 195-bp 3'-UTR (Fig. 1A). Notably, 64.6 % of the ORF exhibited GC content, with over 80 % of this content found in the first 164 bp from the start codon (ATG) and 200 bp downstream (Fig. 1A and B). The 5’-UTR and 3’-UTR exhibited GC content of 78.5 % and 65.4 %, respectively. The complete *Ame-nAChR-a1* gene contained an ORF of 1884 base pairs (Fig. 1C). Within this ORF, two regions of approximately 100 bp, each located at positions 1200 and 1450, exhibited a notably high GC content of nearly 75 % (Fig. 1D).

**Fig. 2:**
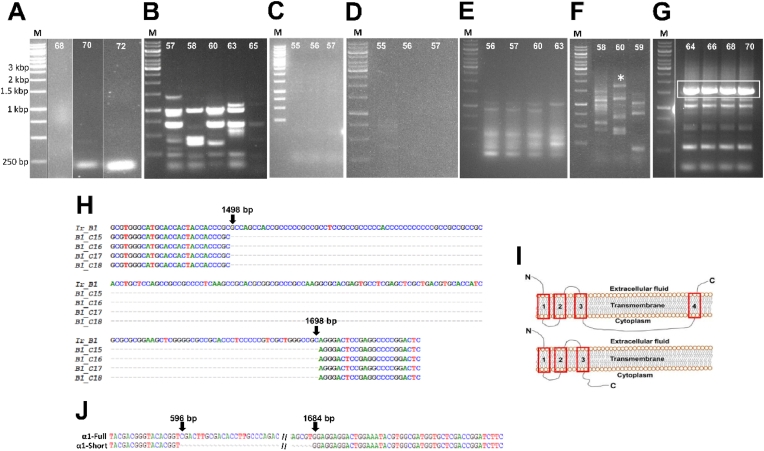
PCR amplification of nAChR subunits with different DNA polymerases: SuperScrip IV One-Step RT-PCR (A–B), DreamTaq DNA Polymerase (C), AccuPrime™ Taq DNA Polymerase (D), Phusion™ High–Fidelity DNA Polymerase (E), and Platinum SuperFi DNA Polymerase amplification of *Ir-nAChRb1* with the primer pair designed for the UTRs (F), or reamplification of the complete ORF of *Ir-nAChRb1* (G) using the PCR template indicated with an asterisk in panel F. Sequencing results of four different plasmids isolated from different bacterial colonies confirmed the *Ir-nAChRb1* sequence with an unamplified 200 bp from >80 % highly GC-rich region (H). The predicted transmembrane domains of the Ir-nAChRb1 subunit are presented in panel I. The full-length Ir-nAChRb1 subunit contained four transmembrane helices between 279 and 563 amino acids (upper part of panel I). The absence of 200 bp from the GC-rich region resulted in a frameshift, leading to an incomplete fourth helix in the translated protein (lower part of panel J). Sequencing analyses of *Ame-nAChR-a1* truncated PCR amplicons showed deletions of 1,086 bases (J).

SuperScript IV One-Step RT-PCR System typically delivers a good PCR yield when amplifying genes directly from RNA samples. However, attempts to amplify the *Ir-nAChRb1* ORF using gradient PCR under standard conditions with cloning primers (primer sets F and R; Fig. 1A and Table 1) failed to produce any product at annealing temperatures of 68 °C and 70 °C (Fig. 2A). Similarly, a two-step PCR protocol combining annealing and extension at 72 °C failed to yield the desired amplicon (Fig. 2A). To improve RT-PCR efficiencies for GC-rich or structurally complex targets, the manufacturer recommends increasing the cDNA synthesis incubation temperature from 50 °C to 55–60 °C. This was attempted, but did not improve the amplification of the genes included in this study (results not shown).

Selecting the primer is crucial for successfully amplifying the gene by PCR. The recommended primer length should be between 15 and 30 base pairs, with a melting temperature ranging from 52 to 58 °C. [27]. However, cloning high-GC-rich genes using PCR with long primers presents additional challenges during amplification due to extra hydrogen bonds between guanine and cytosine bases. [27]. In the *Ir-nAChRb1* ORF, the first 220 bp had a high GC content above 80 %. To mitigate this challenge, we designed primers within regions of the gene with lower GC content to achieve optimal PCR results by keeping the *Tm* of primers between 55 °C and 60 °C. A set of primers (primer sets F1 and R1; Fig. 1A and Table 1) was designed for the UTR regions of *Ir-nAChRb1* and used as a template for a second round of PCR, where longer cloning primers were employed to amplify and clone the complete ORF of *Ir-nAChRb1*. However, this approach failed to yield the desired amplicon, producing nonspecific amplicons (Fig. 2B) at various annealing temperatures (57–65 °C).

Attempts to amplify *Ir-nAChRb1* with Taq DNA polymerases, including DreamTaq (Fig. 2C), AccuPrime Taq (Fig. 2D) and Phusion high-fidelity DNA polymerase (Fig. 2E) were unsuccessful. Notably, Taq DNA polymerases that lack proofreading activity [28] failed to amplify the gene (Fig. 2C and D), whereas Phusion High-Fidelity DNA Polymerase, which has proofreading capability, yielded non-specific amplicons (Fig. 2E). Similarly, we failed to amplify the full-length ORF of *Ame-nAChR-a1* using the Phusion High-Fidelity DNA Polymerase (data not shown). Previous studies have shown a variation in the amplification efficiency of GC-rich genes with different DNA polymerases [18,[29], [30], [31]]. Moreover, it has been shown that proofreading DNA polymerases are more susceptible to failure in amplifying nucleotide sequences high in guanine (G) compared to non-proofreading polymerases [30]. Nevertheless, the proofreading polymerase SuperFi polymerase was found to be effective in most cases to amplify high GC DNA sequences in *Mycobacterium bovis* [18]. In the current study, the amplification of *Ir-nAChRb1* was obtained by Platinum SuperFi DNA Polymerase, although with additional nonspecific PCR products (Fig. 2F). The purified PCR product from *Ir-nAChRb1* (shown by an asterisk in Fig. 2F) was used as a second PCR template to amplify the complete ORF using cloning primers. Subsequently, the purified complete ORF was digested and cloned into a pT7Ts-rich transcription vector. Following isolation, four plasmids were obtained, and sequencing revealed a 200-bp absence of nucleotides 1498 to 1698. For clarity, only the number of nucleotides in the 5'-UTR is shown (Fig. 2H). Additionally, the absence of these bases in PCR led to a frameshift mutation, altering the reading frame of the coding region and resulting in the formation of a truncated protein shorter than the full product size (Fig. 2I, lower panel). Prediction of transmembrane helices in *Ir-nAChRb1* (Fig. 2I) confirmed the absence of a fourth helix, with the C-terminus of the protein ending up in the cytoplasmic region. This is in contrast to the hallmark of nAChR subunits in vertebrates and invertebrates, where both the N-terminus and C-terminus are located on the extracellular side of the cellular membrane [32]. These results suggested that Platinum SuperFi DNA Polymerase was able to partially amplify the *Ir-nAChRb1* but still needed optimization to obtain the complete *Ir-nAChRb1* in RT-PCR.

The honey bee receptor *Ame-nAChR-a* has been studied functionally, although only on an *in vitro* assembled gene [24]. In the current study, initial attempts to amplify the *Ame-nAChR-a* using primer pairs *Ame-nAChR-a1*_F5 and *Ame-nAChR-a1*_R5 (Fig. 1C and Table 1) resulted in multiple bands. After isolating, cloning, and sequencing these PCR products, a deletion of 1086 bp from nucleotides 597 to 1682 was discovered (Fig. 2J). This result was comparable to what was found after amplification of *Ir-nAChRb1*. Proofreading polymerases exhibit higher fidelity and performance compared to non-proofreading polymerases but could not amplify most of the target, for unknown reasons [33,34]. The partial amplification of *Ame-nAChR-a* could be due to the formation of hairpin structures in the GC-rich region, which may cause polymerase dissociation from the template. It is therefore likely possible that impaired amplification leads to the formation of incomplete fragments.

**Fig. 3:**
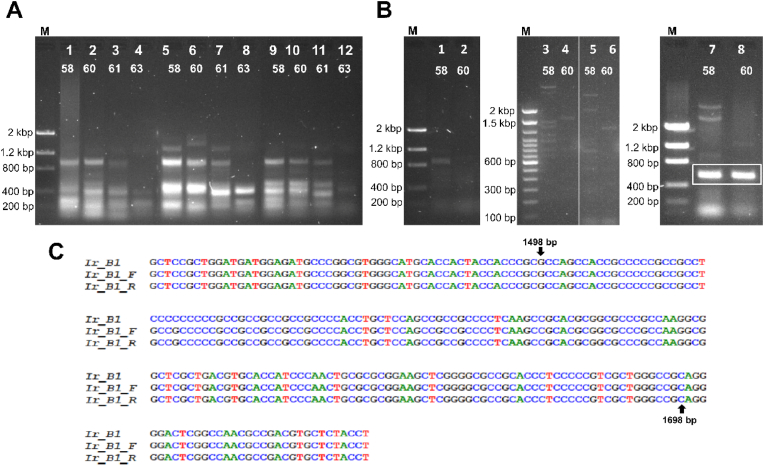
Effect of different additives on cDNA synthesis and PCR (A) Amplification of *Ir-nAChRb1* (583 bp) from cDNA, without additives. Only the additives were included in the PCR. Lane M: DNA ladder; lanes 1–4: 5 % DMSO; lanes 5–8: 1 M betaine; lanes 9–12: 5 % DMSO and 1 M betaine. (B) cDNA synthesis and PCR amplification in the presence of additives. Lane M: DNA ladder; lanes 1–2: No additives; 3–4: 5 % DMSO; lanes 5–6: 1 M betaine; lanes 7–12: 5 % DMSO and 1 M betaine. (C) Sequencing results using forward and reverse primers for PCR products. Combining DMSO and betaine allowed the full amplification of the GC-rich region of 200 bp (black arrows) in both cDNA synthesis and PCR.

**Fig. 4:**
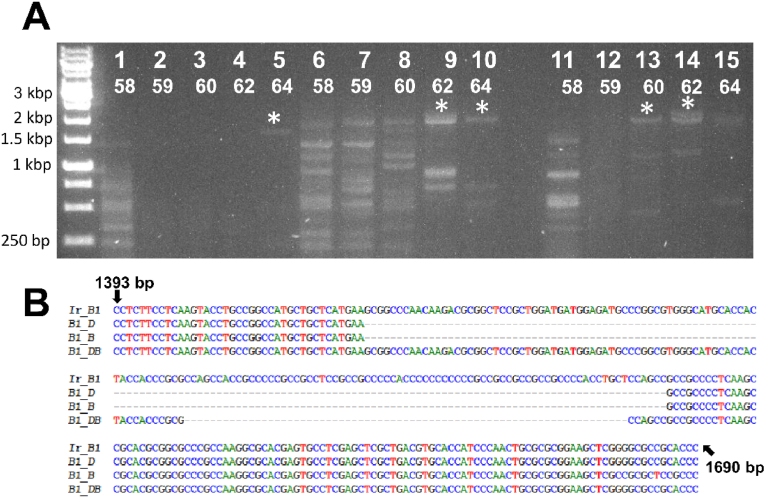
Effects of different additives on amplification of the complete ORF of *Ir-nAChRb1*. (A) cDNA was produced in the absence of additives and PCR was conducted with additives alone or in combination. Lane M: DNA ladder; lanes 1–5: 5 % DMSO; lanes 6–10: 1 M betaine; lanes 11–15: 5 % DMSO and 1 M betaine. (B) Sequencing results from the amplified amplicons are shown with asterisks. Additives in PCR did not amplify the full 200 bp high GC-rich region. Abbreviations: D, DMSO; B, betaine.

Previous studies have proposed different enhancers to improve PCR performance. Among these enhancers are betaine and DMSO, due to their proven efficacy in destabilizing GC-rich secondary structures [7,9,10,35]. Moreover, the addition of betaine and DMSO in PCR improves the yield, reliability, and specificity by reducing the non-specific amplification (For details, see review [36]). While most studies have focused on amplifying GC-rich targets under 1 KB in size, a few have demonstrated success with larger targets when enhancers are used directly in PCR or cDNA synthesis. [6,18,35,37,38]. In our study, we used DMSO and betaine separately or in combination during PCR and cDNA synthesis [2] to facilitate efficient amplification of the complete ORF of *Ir-nAChRb1* and *Ame-nAChR-a1*. First, we attempted to amplify the *Ir-nAChRb1* short fragment of 583 bp (Fig. 3A) containing a high GC-rich 200 bp fragment together with the full-length ORF of both nAChR subunits (Fig. 4) using enhancers in PCR. Initially, the 583 bp fragment was amplified (primer sets DF1 and DR2, Fig. 1A and Table 1) using 5 % DMSO (Lane 1–4), 1 M betaine (Lane 5–8), and an additive mixture (Lane 9–12) (Fig. 3). Platinum SuperFi DNA Polymerase was used in all PCR reactions with the GC enhancer, and cDNA was synthesized without additives. However, PCR mixtures containing different additives failed to produce specific amplicons and yielded multiple undesired products (Fig. 3A). Subsequent synthesis of cDNA in the presence of 2.5 % DMSO and 1 M betaine, followed by PCR amplification under similar conditions (Fig. 3B), resulted in successful amplification only when DMSO and betaine were combined (Lane 7–8, Fig. 3B). Sequencing confirmed that the 200 bases with high GC content were missing (Fig. 2H) when both enhancers were absent in the cDNA synthesis and PCR reaction. These results suggest that the addition of DMSO and betaine in cDNA synthesis and PCR helps in the amplification of *Ir-nAChRb1*, more specifically by disrupting the secondary structures in the GC-rich region.

**Fig. 5:**
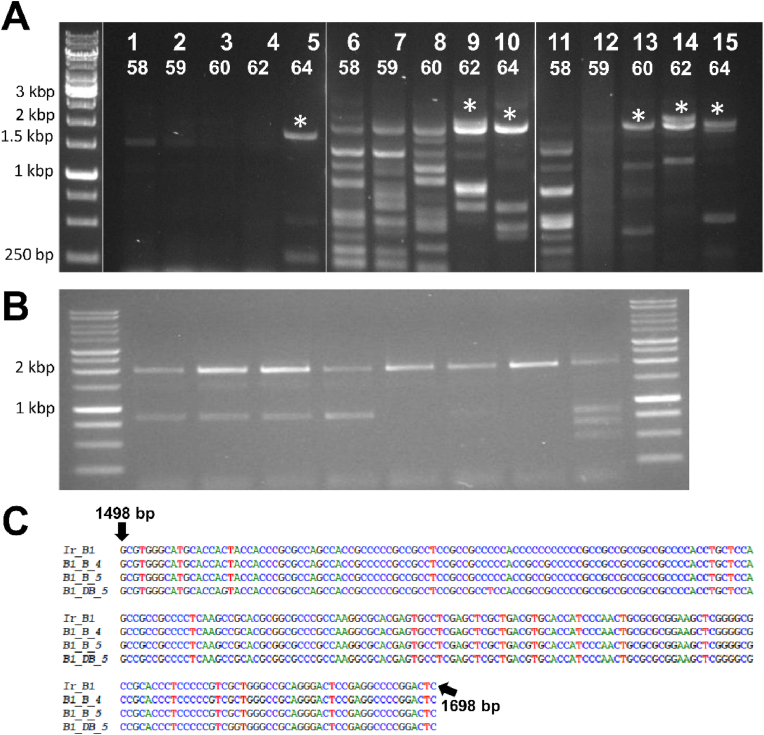
Effects of different additives on amplification of the complete ORF of the nAChR subunits. (A) The complete ORF of *Ir-nAChRb1* was amplified by PCR from cDNA synthesized in the presence of different additives, either alone or in combination. Lane M: DNA ladder; lanes 1–5: 5 % DMSO; lanes 6–10: 1 M betaine; lanes 11–15: 5 % DMSO + 1 M betaine. Asterisks represent expected amplicon sizes. PCR was carried out under different annealing conditions for each additive. (B) Amplification of *Ame-nAChR-a1* with 4 % DMSO alone. Each lane corresponds to the PCR product amplified individually using the same primer pairs and PCR conditions. (C) Sequencing results of PCR products of *Ir-nAChRb1*. Abbreviations: D, DMSO; B, Betaine.

Further attempts to amplify the complete ORF of *Ir-nAChRb1* with and without enhancers were employed. First, PCR was conducted using enhancers but without using enhancers in the cDNA reaction (Fig. 4). PCR revealed intensified bands at 64 °C with the addition of 5 % DMSO to the PCR reaction mix (Fig. 4A). Expected amplicons were observed between 1.5 kb and 2 kb with the addition of 1 M betaine, either alone or in combination with 5 % DMSO. While numerous undesired amplicons were observed with 1 M betaine alone, their occurrence was significantly reduced when betaine was combined with DMSO. Sequencing of the purified PCR products confirmed improved amplification with betaine alone or in combination with DMSO, however the GC-rich region was not fully amplified (Fig. 4B). Similarly, when cDNA was synthesized in the presence of 2.5 % DMSO and 1 M betaine and used as a template in PCR reactions, successful amplification of *Ir-nAChRb1* was achieved only with the addition of both enhancers. The full-length ORF was amplified, including the 200 high GC-rich bases amplified during an initial PCR (Fig. 5A and C). This was confirmed by the sequencing of the amplicons with the expected size. Accordingly, the addition of 4 % DMSO proved crucial in facilitating the amplification of the complete target sequence of *Ame-nAChR-a1* (Fig. 5B) for the first time using the Platinum Taq polymerase, a hybridization temperature of 58 °C, and the primer pair F2 and R1. Each lane (Fig. 5B) corresponds to the PCR product amplified individually using the same primer pairs and PCR conditions. Notably, some replicates exhibited additional smaller bands of approximately 800 bp. Although these bands were not directly sequenced, they likely corresponded to the truncated fragment described earlier, which lacked more than 1,000 bp of the full-length sequence. This observation is consistent with the amplification challenges in GC-rich regions.

The results gained in the current study highlight the enhanced PCR efficiencies for GC-rich targets over 1 KB in size with betaine added alone or in combination with DMSO to the cDNA synthesis and PCR reaction. Although the current study focuses on the amplification of invertebrate genes encoding nAChR subunits, it is highly probable that the same strategy will be successful for other groups of genes with high content of GC in specific regions.

## Author contributions

Conceptualization: MTK Methodology: MTK, LR, CB Validation: MTK, LR, MJB, TEH Formal analysis: MTK, LR, CB Investigation: MTK, LR Data curation: MTK, LR, TEH, MJB Writing original draft: MTK; LR, Writing review and editing: MTK; LR, TEH, MJB Visualization: MTK, LR Supervision: MJB, TEH Funding acquisition: TEH, MJB.

## Funding

This work was supported by the Research Council of Norway, Grant Number 325190. The Research Council of Norway had no role in study design, data collection and analysis, manuscript preparation or publication decisions.

## Declaration of competing interest

The authors declare that they do not have any conflict of interest.

## Data Availability

No data was used for the research described in the article.
